# Obesity and Cancer Progression: Is There a Role of Fatty Acid Metabolism?

**DOI:** 10.1155/2015/274585

**Published:** 2015-03-19

**Authors:** Seher Balaban, Lisa S. Lee, Mark Schreuder, Andrew J. Hoy

**Affiliations:** ^1^Discipline of Physiology, School of Medical Sciences and Bosch Institute, The University of Sydney, Sydney, NSW 2006, Australia; ^2^Charles Perkins Centre, The University of Sydney, Sydney, NSW 2006, Australia; ^3^Boden Institute of Obesity, Nutrition, Exercise & Eating Disorders, The University of Sydney, Sydney, NSW 2006, Australia

## Abstract

Currently, there is renewed interest in elucidating the metabolic characteristics of cancer and how these characteristics may be exploited as therapeutic targets. Much attention has centered on glucose, glutamine and *de novo* lipogenesis, yet the metabolism of fatty acids that arise from extracellular, as well as intracellular, stores as triacylglycerol has received much less attention. This review focuses on the key pathways of fatty acid metabolism, including uptake, esterification, lipolysis, and mitochondrial oxidation, and how the regulators of these pathways are altered in cancer. Additionally, we discuss the potential link that fatty acid metabolism may serve between obesity and changes in cancer progression.

## 1. Introduction

Obesity has long been known to be associated with the development of type 2 diabetes and cardiovascular disease [1]. More recently there is a growing acceptance for a link between obesity and cancer [2]. However, the nature of this relationship remains to be fully elucidated. On one hand obesity increases the risk of many types of cancer, including esophageal, endometrial, thyroid, colon, renal, liver, and breast [3, 4]. The other aspect is that obesity is also associated with changes in the progression of many cancers. These include higher grade disease in prostate and breast cancer [5, 6] and poorer outcomes in endometrial, kidney, pancreas, esophageal, and thyroid cancers [7–9].

**Figure 1:**
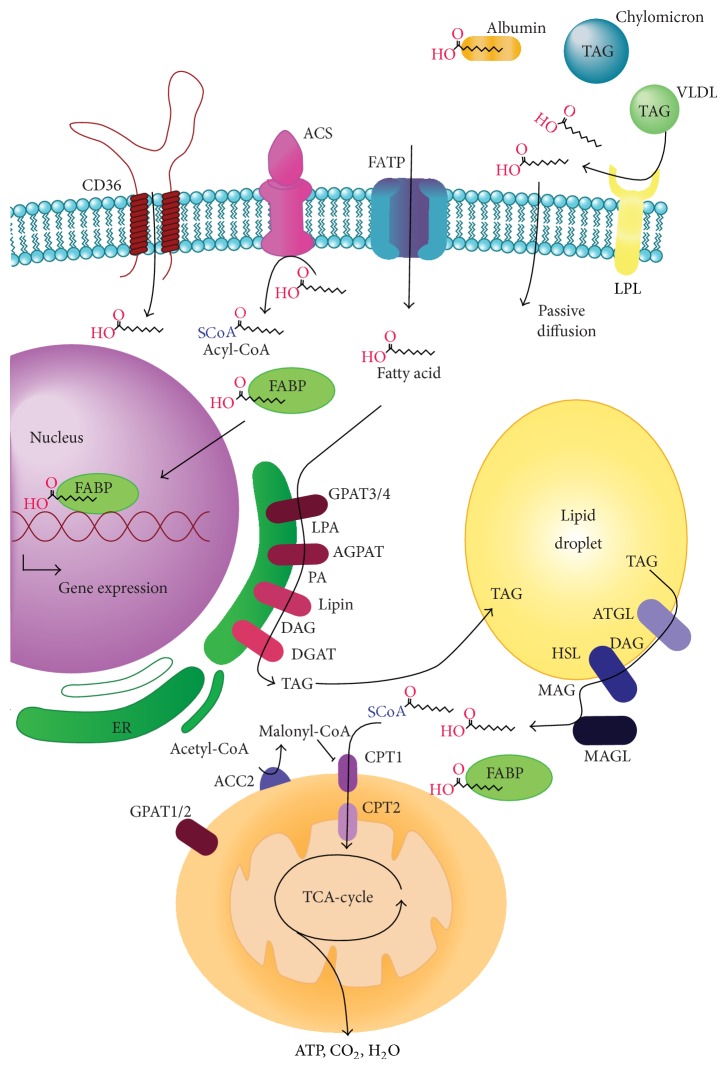
Intracellular fatty acid metabolism. A simplified cartoon of fatty acid metabolism pathways. Fatty acids are transported in the circulation as triacylglycerol (TAG) in lipoproteins and hydrolyzed by lipoprotein lipase (LPL) or they are bound to albumin and are transported across the plasma membrane. A CoA is ligated to fatty acid (FA), and the fatty acyl-CoA (FA-CoA) can enter the glycerolipid synthesis pathway for storage or the mitochondria for oxidation. ACS, acyl-CoA synthase; AGPAT, acyl-CoA: acylglycerol-3-phosphate acyltransferase; ATGL, adipose triglyceride lipase; DAG, diacylglycerol; DGAT, diacylglycerol acyltransferase; GPAT, glycerol-3-phosphate acyltransferase; HSL, hormone-sensitive lipase; LPA, lysophosphatidic acid; MAG, monoacylglycerol; MAGL, monoacylglycerol lipase; PA, phosphatidic acid.

Obesity is defined by increased adipose mass arising from energy imbalance. The predominant cell type in adipose tissue is the adipocyte, which is the professional lipid storage cell. Alongside the adipocyte there are a number of other cell types in adipose including preadipocytes, endothelial cells, and immune cells such as resident macrophages. This collective results in a highly complex organ that is central to energy homeostasis and its biology is dramatically altered in obesity. These changes include altered adipocyte biology, such as increased efflux of fatty acids and modified adipokine profile, which is often accompanied by low-grade inflammation and hyperinsulinemia [10]. Whilst these changes are common, they are not defining characteristics of the entire obese population. For example, a significant subpopulation is metabolically healthy, retains insulin sensitivity, and has normal lipid and inflammation profiles [11]. Likewise, there are other populations, including the “metabolically obese, normal weight” [12, 13] and those with familial lipodystrophy [14, 15] that have pathogenic metabolic profiles. These other populations highlight that inflammatory mediators and increased growth factor availability (e.g., IGF-1, insulin; see [16]) are not the only mechanisms linking obesity with cancer. In this review, we will highlight the evidence that exists on the role that fatty acid metabolism plays in cancer biology (Table 1), focusing on pathways of fatty acid uptake, storage, mobilization, and oxidation (Figure 1). This focus is based upon the potential link that fatty acid metabolism may play in the obesity/cancer relationship as excessive lipid accumulation, particularly in abdominal regions, is a definitive characteristic of obesity.

## 2. Lipoprotein Hydrolysis, Fatty Acid Transport, and Trafficking

Long-chain fatty acids travel in the circulation either as free fatty acids that are released from adipocytes bound to albumin or as triacylglycerol (TAG) contained in very low-density lipoproteins and chylomicrons. This circulating TAG is hydrolyzed by lipoprotein lipase (LPL) to free fatty acids [68] and then taken up into cells (Figure 1). There remains some controversy as to whether these fatty acids enter the cell by passive diffusion or by protein mediated transport. As will be discussed below, it is clear that the latter process does contribute to fatty acid uptake.

### 2.1. Lipoprotein Hydrolysis

Altered expression of LPL has been reported in many cancers. For example, Narita and colleagues [17] reported a significant association between increased hydrolytic activity of LPL due to the LPL polymorphism (Ser447stop) and the susceptibility to prostate cancer. This association was even stronger in patients with high grade tumors or metastasis. Similarly, this pattern was observed in cervical cancer where LPL is frequently overexpressed in cervical squamous cell carcinomas and associated with an increased invasion capacity [18]. LPL activity has been reported in gastric and rectal cancers, malignant fibrous histiocytomas, and osteosarcomas, with the high proliferating outer area of rectal tumors and fibrous histiocytomas having an enhanced expression of LPL compared with the center [19]. Interestingly, the increased LPL activity in cancer tissue, compared with healthy lung tissue, predicts lower overall survival in non-small-cell lung cancer [20, 21]. The location of tumor LPL is somewhat controversial as a recent study observed that increased LPL expression was in a subgroup of macrophages and not in cancer cells [69]. These studies mostly report gene expression and therefore future studies linking functional changes in cancer cell LPL activity driving FA release from circulating TAG are required, especially as LPL activity is regulated by a variety of physiological stimuli (see review [70]).

### 2.2. Fatty Acid Transport

Several proteins have been identified to facilitate the uptake of fatty acids into cells. These include CD36/fatty acid translocase, the fatty acid binding protein (FABP) family, and the fatty acid transport proteins (FATP) [71]. Many of these transporters are ubiquitously expressed, while some display tissue-specific expression [72, 73]. Interestingly, most tissues have coexpression of different fatty acid transporters [74]. The reason for this remains unknown. Possibilities may include differences in uptake capacity and substrate specificity, sensitivity to hormonal stimuli such as insulin [75], or preferences in partitioning into downstream pathway, for example, fatty acid esterification (storage) or oxidation [74].

#### 2.2.1. CD36/Fatty Acid Translocase

CD36, also known as fatty acid translocase (FAT), is a multifunctional transmembrane glycoprotein which is abundantly expressed in cell types active in fatty acid metabolism, including adipocytes, skeletal muscles cells, cardiomyocytes, intestinal enterocytes, monocytes, and hepatocytes [76]. It was originally isolated from platelet membranes as a thrombospondin receptor [77] but has also been shown as a receptor for collagen [78], oxidized lipoproteins [79], and, of greatest interest to this review, long-chain fatty acids [80].

CD36 has been implicated in contributing to cancer progression. Low CD36 gene expression correlates with a higher metastasis grade in colon and ovarian cancers and with low recurrence-free survival [22]. Conversely, CD36 mRNA expression in breast cancer is inversely correlated with the metastatic potential of five breast cancer cell lines [23], where its expression is relatively higher in less aggressive cell lines (T47-D and MCF-7) and almost absent in highly aggressive lines (ZR-75 and MDA-MB-231). This inconsistency between cancer types may be explained by the multifunctionality of CD36. While it functions as a fatty acid transporter, CD36 is also involved in collagen adhesion and, therefore, less CD36 may reduce cell adhesion, providing cancer cells with a higher metastatic potential. That said, the above studies have reported gene or protein expression and not the rates of fatty acid uptake.

Fatty acid transporter abundance is not the only factor regulating FA uptake. An aspect that is often overlooked is that FA uptake is increased by insulin stimulation [81, 82]. This is thought to be mediated by translocation of CD36 to the plasma membrane which has been observed in hepatocytes of obese Zucker rats [83], skeletal muscle [84, 85], cardiomyocytes [86, 87], and ovary cells [85]. This is analogous to the translocation of the insulin sensitive glucose transporter GLUT4 [88, 89].

So far, no studies have investigated the influence of obesity on fatty acid transporters in cancer cells. It is clear from studies in other model systems that CD36 expression and fatty acid uptake are influenced by the microenvironment. For example, CD36 gene expression and protein levels are increased in steatotic hepatocytes [90] and liver biopsies of obese patients, correlating with the circulating free fatty acids levels [91]. In subcutaneous adipose tissue, CD36 protein expression is upregulated in both obese patients and type 2 diabetics [92]. Furthermore, CD36 mRNA expression levels are greatly enhanced in liver and adipose tissue of* ob/ob* mice, a monogenic model of obesity [93]. Interestingly, incubation of human skeletal muscle cells with adipocyte conditioned media increased both fatty acid uptake and CD36 protein levels [94]. Similar changes in CD36 expression by adipocyte factors, such as adipokines and fatty acids, have been reported in vascular smooth muscle cells [95], cardiomyocytes [86, 96], and adipocytes [97, 98]. Collectively this suggests that changes in adipocyte biology, especially in the context of obesity, can alter CD36 expression in nonadipose cells such as cancer cells that may influence the inherent role that CD36 plays in cancer biology.

#### 2.2.2. Fatty Acid Transport Protein

Fatty acid transport proteins form a highly conserved family of six transporters named FATP1–6 [99]. FATPs are integral membrane proteins and are differentially expressed in a wide variety of cells [100]. These transporters are unique as they can express fatty acyl-CoA synthetase activity [101] as well as an endoplasmic reticulum localization signal domain, at least for FATP4 [102]. Alongside CD36, FATPs regulate long-chain fatty acid and very long-chain fatty acid uptake [103] although the functional differences between CD36 and FATPs are yet to be resolved. A recent study in Madin-Darby Canine cells reported that CD36 is 30-fold more effective in fatty acid uptake compared with FATP4 or the acyl-CoA synthetase ACSL1 [104]. However, cooverexpression of CD36 with either FATP4 or ACSL1 results in an enhanced fatty acid uptake rate greater than expected from the combined individual capacity suggesting a synergistic relationship between CD36, FATP4, and ACSL1 to facilitate fatty acid uptake.

To date, only one study has described a possible role for FATPs in tumor metabolism. In this study, FATP mRNA expression is increased in rat hepatomas compared with normal liver tissue which correlated with fatty acid uptake rates [24]. Similar to CD36, FATP expression is influenced by the microenvironment, especially in obesity. FATP expression is elevated in adipose tissue of obese patients [105, 106] and in heart [107], skeletal muscle [108], and adipose tissue [109] of rodent models of obesity. Overall, FATPs are important players in lipid uptake and metabolism. However, their role in cancer, especially in the context of obesity-sensitive cancers, is far from understood and further research is needed to elucidate this role.

### 2.3. Intracellular Trafficking

Fatty acid binding proteins (FABPs) are a family of transport proteins with high affinity for long-chain fatty acids, bile acids, and retinoids [110]. Twelve FABP isoforms have been identified, each with its own tissue and substrate specificity [111]. Although their physiological functions are not fully understood, they appear to facilitate the transport of fatty acids intracellularly and thereby regulate substrate availability for complex lipid synthesis (esterification) and oxidation [112, 113]. Changes in FABP expression have been associated with various diseases including several forms of cancer [113] with FABP5 being the most well characterized FABP isoform in cancer cell biology. For example, prostate [32], endometrial [34], liver [35], pancreatic [36], and breast [32] cancers have increased FABP5 gene or protein expression. However, the observations in prostate are controversial as other studies report reduced expression in multiple prostate cancer lines [26, 38]. Despite this, increased expression of FABP5 in prostate cancer cells increased fatty acid uptake and peroxisome proliferator-activated receptor gamma (PPAR*γ*) expression which enhanced tumor progression [37]. Additionally, overexpression of FABP5 in the benign breast cancer cell line, Rama 37, increased metastatic capacity in rats [32]. Interestingly, expression is higher in estrogen and progesterone negative breast cancer cells, with the highest expression found in triple-negative breast cancer [33]. Furthermore, patients with higher FABP5 mRNA levels had lower recurrence-free and overall survival probabilities [33]. Conversely, invasion capacity and tumor growth were significantly reduced in prostate cancer cells with reduced FABP5 expression [39].

FABP7 has emerged as another participant of intracellular FA metabolism that may contribute to cancer cell biology. Its gene expression is elevated in triple-negative breast cancer cells [40], primary melanomas [43], and renal cell carcinomas [44, 45]. Interestingly and in contrast to FABP5, FABP7 positive basal-like breast tumors had a significant lower recurrence rate and improved survival rate [41]. In cell culture studies, siRNA knockdown of FABP7 reduced proliferation and invasion in melanoma cells whilst the contraobservation was reported with overexpression enhancing proliferation and invasion [114]. Furthermore, investigations of the organelle-specific roles of FABP7 demonstrated that increased nuclear, but not cytoplasmic, FABP7 is associated with increased proliferation, pleomorphism, and tumor stage in breast cancer suggesting that nuclear FABP7 drives a more aggressive phenotype [42]. However, the mechanism by which FABP7 influences gene expression is yet to be resolved. FABPs may act as coactivators for transcription factors like PPARs [115] or simply function as transporters to carry FA into the nucleus to modulate gene expression [116] via the many intranuclear targets including sterol regulatory binding protein, PPARs, and liver X receptors [117].

FABP4 has also been implicated in cancer biology. FABP4 mRNA levels are downregulated in breast cancer cells [25]. Conversely, FABP4 expression is inversely correlated with tumor progression and invasiveness in bladder cancer [26–29]. FAPB4 is also susceptible to the extracellular milieu as there is growing evidence that adipocytes increase FABP4 mRNA and protein expression in cancer cells. An elegant study in ovarian cancer demonstrated that coculture with adipocytes increases FABP4 protein expression and promotes migration and invasion of ovarian cancer cells, while FABP4 deficiency ameliorated the adipocyte-derived metastatic potential [31]. A similar observation of adipocyte-induced increase in FABP4 expression has been reported in PC3 prostate cancer cells [30]. The same study also reported an increased expression of FABP4 in prostate cancer bone metastasis from high-fat diet mice and prostate cancer patients [30]. The fact that bone marrow is adipocyte-rich [118] suggests a role for adipocytes in enhancing FABP4 expression and thereby playing an important role in cancer progression. Overall, FABPs are emerging as important factors in cancer cell lipid metabolism but more research is needed to fully elucidate the roles of FABPs in healthy tissue and tumor cells and how these are altered by obesity.

## 3. Fatty Acid Activation, Esterification, and Mobilization

Once FAs are taken up by cells, they are activated by the addition of coenzyme A (CoA) to the fatty acid molecule by the actions of long-chain acyl-CoA synthetase (ACSL). From here, evidence suggests that fatty acyl-CoAs can be partitioned into the esterification pathway in the endoplasmic reticulum or the mitochondria for oxidation [119]. Recently, this notion has been challenged by studies in human skeletal muscle [120] and isolated hepatocytes from mice lacking adipose triglyceride lipase (ATGL) [121]. These studies suggest that extracellular FAs enter the esterification pathway to be stored in lipid droplets prior to mitochondrial oxidation. Irrespective of the precise pathways, fatty acids have a multitude of intracellular fates, but at the most basic level FAs can be either oxidized or stored as complex lipids.

### 3.1. Fatty Acid Activation

ACSLs are a family of enzymes that catalyze the addition of a CoA to a free fatty acid and differ in their preference to the chain length of their fatty acids substrates (short, medium, long, and very long). ACSL1, ACSL3, ACSL4, ACSL5, and ACSL6 are members of the long-chain family that vary in both subcellular localization and substrate specificity [122]. Along with FABPs, individual ACSL isoforms have been proposed to channel fatty acids to specific metabolic pathways.

Significant evidence suggests an important role for ACSLs in cancer biology including increased expression of ACSLs in many types of cancer such as colon, liver, lung, brain, and colorectal cancers and estrogen receptor negative breast tumors and androgen receptor negative prostate tumors [46, 47, 49, 123–125]. More specifically, ACSL5 gene expression is consistently elevated in the colon cancer tissue compared to normal colon tissue [49], so are ACSL4 gene expression and protein levels in colon adenocarcinoma compared with adjacent normal tissue [47] and in hepatocellular carcinoma tissues compared to the adjacent noncancerous liver tissue [48]. Finally, ACSL3 expression is elevated in the highly tumorigenic U87 human glioblastoma cell line and cells derived from tumorigenic primary glioblastoma xenografts (Mayo 22) compared with the less tumorigenic U373 glioma cells [46].

Collectively, the results from these studies suggest that expression of ACSLs is related to tumorigenesis and tumor progression. Cell culture loss and gain of function studies provide insight into the relationship between altered intracellular fatty acid metabolism and cancer cell biology. In terms of fatty acid metabolism, both ACSL3 and ACSL5 overexpression in HepG2 cells increase fatty acid oxidation and reduce TAG levels [126]. Supporting the gene expression observations, altered ASCL expression in cancer cells is linked with survival, proliferation, and chemoresistance. For example, overexpressed ACSL4 in human epithelial cells reduced the level of arachidonic acid-induced apoptosis [127], whereas siRNA-mediated ACSL3 knockdown reduced growth rates of lung cancer cell lines and colony formation [124]. Similarly, ACSL4 knockdown inhibited growth rates of the human hepatocellular carcinoma cell line Hep3B [48]. Additionally, pharmacological inhibition of ACS activity by triacsin-C induced apoptosis in HEK293 cells [127] and glioma cells, which was completely suppressed by overexpression of ACSL5 [128].

The impact of ASCL expression and function in cancer biology in the obese setting has not been reported. Interestingly, ACSL activity and* Acsl1* gene expression are upregulated in liver and adipose tissues in genetic obese models, including ob/ob mice and Zucker fatty rat (fa/fa) [93, 129] and high-fat fed rats [130]. This suggests that the elevated expression reported may be exacerbated in obesity and therefore may accelerate cancer progression. How changes in ASCL-mediated fatty acid metabolism are linked to altered cancer progression is yet to be fully elucidated. However, Cao and colleagues [127] proposed that changes in proapoptotic arachidonic acid levels may play a role yet other bioactive lipids such as sphingolipids, including ceramides, or changes in fatty acyl-CoA availability for mitochondrial oxidation are potential contributors.

### 3.2. Fatty Acid Esterification

FAs are the building blocks for many complex lipids including phospholipids, sphingolipids, and glycerolipids. We will focus on the synthesis of glycerolipids, such as TAG, as this is a major pool that is susceptible to the obese environment. The storage of fatty acids as TAG involves several condensation reactions. The first step involves esterifying a fatty acyl-CoA with glycerol-3-phosphate to generate lysophosphatidic acid (LPA) by the enzyme glycerol-3-phosphate acyltransferase (GPAT). LPA is then condensed into phosphatidic acid (PA) by 1-acylglycerol-3-phosphate-O-acyltransferase (AGPAT). The subsequent reaction is catalyzed by lipin, which dephosphorylates PA to produce diacylglycerol (DAG). The final step involves the addition of a third fatty acyl-CoA to DAG by diacylglycerol acyltransferase (DGAT) to generate TAG. This process occurs in the endoplasmic reticulum where TAG is packaged into lipid droplets [131]. Alongside the endoplasmic reticulum pathway, there is evidence that DGAT can also catalyze the conversion of DAG to TAG at the lipid droplet [132–134].

The lipid intermediates of the esterification pathway are substrates for the generation of other complex lipids, such as phospholipids in membrane synthesis, and can also act as lipid signals that modify membrane structures and promote gene transcription for cell growth, proliferation, and differentiation [135].

To date, gene or protein expression profiling of GPAT in cancer cells has not been reported. However, it is known that four isoforms of GPATs are expressed in mammals; GPAT1 and GPAT2 are localized in the mitochondria and GPAT3 and GPAT4 in the endoplasmic reticulum [136]. As rate-limiting enzymes of fatty acid esterification, GPATs are key regulators of TAG synthesis [137, 138].

Similarly, little is known about the expression of lipin and DGAT in cancer patients. Mammals have three lipin proteins and two isoforms of DGAT that regulate phospholipid synthesis and lipid storage [139, 140]. Consequently, these proteins modulate the availability of fatty acid substrates for lipid signaling and metabolism, which may influence cancer progression [141].

The most studied enzyme of lipid esterification in relation to cancer is 1-acylglycerol-3-phosphate-O-acyltransferase (AGPAT). There are 11 known isoforms of AGPAT, which differ by tissue expression and enzymatic activity [137]. There is consistent evidence suggesting a role for AGPATs in cancer cells. For example, AGPAT2 expression is elevated in ovarian cancer patients with aggressive ovarian cancers and associated with reduced overall survival [50–52]. Gene expression of AGPAT11 is also increased in breast and cervical cancers, as well as colorectal cancer [53]. Interestingly, transcriptional expression of AGPAT9, which is highly homologous with AGPAT11, is upregulated in colorectal cancer, but not in breast and cervical cancers [54].

Obesity is characterized by increased levels of TAG stored in tissues such as skeletal muscle, liver, and cardiac muscle [142], which is a consequence of increased esterification rates [130, 143–145]. Loss and gain of function studies in various tissues provide an insight into the complex regulation of the intracellular lipid environment. For example, AGPAT6 knockout mice have reduced TAG content in brown and white adipose tissue and interestingly altered fatty acid profile of complex lipids, such as DAG and phospholipids with a shift towards the polyunsaturated more than the monounsaturated fatty acids [146]. Similarly, adipose tissue TAG levels are decreased in mice lacking DGAT [147] and both lipin1 and lipin3 [148] and protection from high-fat diet induced obesity and associated metabolic perturbations [139, 149]. Finally, GPAT-deficient mice have lower levels of liver and plasma TAG [150]. On the other hand, overexpressions of GPAT1 [151], GPAT4 [152], AGPAT1 [153], lipin1 [154], and DGAT1 [155] all result in increased TAG levels. From this, it is evident that enzymes involved in esterification significantly influence intracellular and extracellular lipid homeostasis. How this translates to pathogenic changes in cancer cells is yet to be described.

### 3.3. Lipolysis

TAGs, along with cholesterol esters, are stored in lipid droplets to serve as a readily available source of energy for ATP generation in the mitochondria, as well as providing building blocks for phospholipids and other complex lipids. In terms of metabolic energy capacity, an average nonobese person stores up to 2,500 kJ of metabolic energy in glycogen, but >500,000 kJ as TAGs [156]. Whilst most of this TAG is stored in adipocytes, all cells have the capacity to synthesize and breakdown TAGs. Interestingly, intracellular lipid stores, or lipid droplet size and/or number, are elevated in various malignant cells, such as breast [157], prostate [158], cervical [159], liver [160], and colon cancer cells [161]. Furthermore, biochemical assessment of lipid droplets in breast cancer cells has shown that the TAG content is increased [162]. Not only that, TAG levels are higher in more aggressive breast cancer cells and are associated with long-term breast cancer cell survival [157, 162]. These findings suggest that intracellular TAG may play a critical, yet unexplored, role in supporting both substrates for complex lipid synthesis [163] as well as energy production in cancer cells that collectively promote cell growth and proliferation. To do this, TAGs need to be broken down to FAs and glycerol by a process called lipolysis.

ATGL, otherwise known as desnutrin [164], is the predominant TAG lipase that is thought to be rate-limiting [165]. It catalyzes the conversion of TAG to DAG and releases a free fatty acid from the sn-2 position [166]. Hormone-sensitive lipase (HSL) catalyzes the hydrolysis of DAG into monoacylglycerol (MAG) and a fatty acid [167]. HSL has broad substrate specificity, including TAG, DAG, MAG, and cholesterol ester lipid classes, but has the highest affinity for DAG [168]. MAG is then broken down by monoacylglycerol lipase (MAGL) resulting in the metabolic end-product, glycerol, and the liberation of the final fatty acid. This process is highly conserved across species and highly regulated with most insight arising from studies in adipocytes (see review [169]).

Adipose neutral lipase expression in various cancer patients has been reported. Compared to normal individuals without cancer, HSL mRNA expression is elevated in adipose tissue of colorectal, pancreatic, esophageal, and stomach cancer patients [57]. This was also observed in ovarian cancer patients, where adipocyte lipid depots which contain TAG were reduced, while the lipolytic products, MAG and free fatty acids, were increased, collectively suggesting elevated lipolytic activity [170]. Similarly, transcriptional and protein expression of HSL are increased in the adipose tissue of late-stage cancer patients exhibiting uncontrolled loss of adipose and muscle tissue, known as cachexia [55]. Interestingly, upregulated ATGL activity in adipose tissue was found to be responsible for this tissue-wasting syndrome [56].

MAGL is currently the most well-documented neutral lipase and its transcriptional expression is altered in several different cancers. For example, high mRNA expression of MAGL has been reported in ovarian [59], colorectal [58], breast, and melanoma cancer cells and particularly in aggressive prostate cancer cell lines [59]. Interestingly,* in vitro* studies overexpressing MAGL in nonaggressive ovarian cancer cells raised free FA levels and increased tumor growth rate, migration, and invasion [171]. Alternatively, pharmacological inhibition attenuated MAGL-induced aggressiveness of prostate cancer cells, even in a high-lipid environment [59, 171]. Similar observations have been made in colorectal cancer cells [58].

There are conflicting observations regarding the expression patterns of lipolytic enzymes in obesity [172]. HSL and ATGL gene expression are reduced in the adipose tissue of obese humans [173–175] and insulin resistant high-fat fed rats [176]. Conversely, a study by de Naeyer and colleagues [172] has reported that HSL and ATGL mRNA expression are increased in visceral adipose tissue of morbidly obese men; however, this pattern did not translate to changes in protein or lipase activity. This is not surprising considering that these neutral lipases are predominantly regulated by posttranslational modifications, translocation, and protein-protein interactions [135]. Interestingly, lipolytic enzyme expression, particularly ATGL, appears to be more associated with insulin sensitivity rather than obesity [177]. In order to elucidate the role lipolysis plays in cancer cell biology, future studies need to assess pathway of lipid metabolism and fatty acid flux, rather than gene expression, and investigate how these are altered with obesity.

## 4. Mitochondrial Fatty Acid Oxidation

### 4.1. Fatty Acid Entry into the Mitochondria

The other major fate for extracellular fatty acids is oxidation for the generation of ATP in the mitochondria. Alongside glucose and glutamine, fatty acids are a major energy source catabolized through the *β*-oxidation pathway to generate acetyl-CoA for entry into the TCA cycle as well as FADH_2_ and NADH reducing equivalents for use by the electron transport chain (ETC).

Changes in cancer cell fatty acid oxidation have been reported. The primary example is observed in prostate cancer. Rather than being secreted as it is in normal prostate cells, citrate is catabolized in the TCA cycle resulting in fatty acid oxidation being the dominant bioenergetic pathway [178]. Interestingly, high-fat feeding of the p48-Kras mouse model of pancreatic cancer accelerated tumor growth and increased energy expenditure and whole body fatty acid oxidation through increased gene expression of CPT1A, ACC, and AOX enzymes, key regulators of fatty acid oxidation [179].

Few other studies have investigated the effect of obesity on cancer fatty acid oxidation. Although there is significant controversy as to the effect that obesity has on fatty acid oxidation in type 2 diabetes, the increased availability of circulating and intracellular fatty acids is thought to drive an increased oxidative capacity. Evidence for this arises from studies in rodents fed a high-fat diet [124, 180] and obese type II diabetic patients [181]. Conversely, a number of studies reported a reduced capacity to oxidize fatty acids in overweight/obese humans [182, 183]. Considering the high metabolic flexibility of cancer cells, it is conceivable that cancer cells benefit from high lipid availability that characterizes obesity through beta-oxidation either to fulfill increased energy demand or to prevent the lipotoxic effects of high level of fatty acids.

### 4.2. Carnitine Palmitoyltransferase 1

Unlike short-chain fatty acids, which can freely diffuse into mitochondria, long-chain fatty acids enter the mitochondria by the carnitine shuttle system. First, carnitine palmitoyltransferase 1 (CPT1) catalyzes the transfer of the fatty acid moiety from acyl-coenzyme A (CoA) to a long-chain acyl-carnitine. This is then transported into the mitochondrial matrix by the carnitine acyl-carnitine translocase (CACT) [184]. CPT2 then catalyzes the conversion of acyl-carnitine to carnitine and fatty acyl-CoA which then enters the *β*-oxidation pathway. CPT1 is regulated by a cytosolic pool of malonyl-CoA produced by acetyl-CoA carboxylase 2 (ACC2) at the mitochondrial membrane [185].

The rate of mitochondrial fatty acid oxidation is regulated by CPT1, which is an integral membrane protein located on the mitochondrial outer membrane [186]. CPT1 has three isoforms with tissue-specific expressions and sensitivity to the allosteric-inhibitory action of malonyl-CoA: CPT1A (liver), CPT1B (muscle), and CPT1C (brain) [187, 188]. Changes in CPT1 expression have been observed in several types of cancer including breast, lung, brain, and liver cancers [60, 62, 189, 190]. A study by Linher-Melville and colleagues [60] reported that* CPT1A* mRNA levels are significantly elevated in both MCF-7 and MDA-MB-231 cells compared to 184B5 human mammary epithelial cells. In another study,* CPT1C* gene expression is upregulated in non-small-cell lung carcinoma tumor tissue compared with matched normal lung tissue [62]. Furthermore, high grade glioblastoma is associated with increased mRNA levels of both* CPT1A* and* CPT1C* [61]. These studies clearly show that CPT1 expression levels are related to not only tumorigenesis but also tumor progression. In contrast, CPT1 expression has been reported to be higher in the low metastatic potential, androgen receptor negative LNCaP prostate cancer cell line compared to the high metastatic potential, androgen receptor positive PC3 and DU145 prostate cancer cell lines [59]. Overexpression of CPT1C in MCF-7 cell line elevated fatty acid oxidation and ATP production to support resistance to glucose deprivation and siRNA-mediated CPT1C knockdown suppressed xenograft tumor growth [62]. Further evidence for a role for CPT1 in cancer biology has been generated from pharmacological studies. Inhibition of CPT1 with either genetic or pharmacological manipulation has been shown to reduce total ATP levels and the rate of ATP production in PC3 prostate cancer cells [62], Burkitt's lymphoma cells [191], and human glioblastoma cells [192] to impair proliferation. Additionally, etomoxir sensitizes human leukemia cells to apoptosis [193]. Collectively, these studies suggest a role for altered CPT1 expression in various cancers but interestingly CPT1 expression is sensitive to the microenvironment. For example,* CPT1A* mRNA expression and fatty acid oxidation are increased in SKOV3ip1 ovarian cancer cells cocultured with adipocytes [31]. However, it must be highlighted that fatty acid oxidation is regulated at a number of levels including CPT1 gene expression, allosterically by malonyl-CoA and fatty acid availability.

### 4.3. Acetyl-CoA Carboxylase

Acetyl-CoA carboxylase is a biotin-dependent enzyme that catalyzes the conversion of acetyl-CoA into malonyl-CoA. In mammals, two isoforms of ACC are expressed: ACC1 (also known as ACC*α*) and ACC2 (also known as ACC*β*) [194]. ACC1 is primarily expressed in the cytosol of hepatocytes, adipocytes, and other lipogenic cells, while ACC2 is an enzyme associated with the outer mitochondrial membrane and is mainly expressed in cardiomyocytes, skeletal muscles, and hepatocytes [195–198]. Whereas the malonyl-CoA generated by ACC1 is primarily used for* de novo* lipogenesis, the malonyl-CoA product of ACC2 is a potent regulator of fatty acid oxidation by inhibiting CPT1 [199, 200]. Upstream of ACC2 is AMP-activated protein kinase (AMPK), which phosphorylates and inactivates ACC2 to reduce malonyl-CoA levels and thereby increase fatty acid oxidation.

Upregulation of ACC1 and increased* de novo* lipogenesis are observed in several types of cancer including breast [63, 64], prostate [65], lung [66], and liver cancers [67]. Chemical and genetic inhibition studies have identified a role for ACC1 in cell survival. For example, apoptotic cell death results from chemical inhibition of ACC1 by TOFA (5-tetradecyloxy-2-furoic acid) in lung and colon cancer cells [201] and by soraphen A in prostate cancer cells [202]. In addition, RNA interference- (RNAi-) mediated knockdown of ACC1 induces apoptosis in breast [203] and prostate cancer cells [204].

To date, studies have focused on ACC1, yet few studies have been conducted into the role of ACC2 in cancer development or progression. One of these studies demonstrated that knockdown of ACC2 increased fatty acid oxidation and inhibited cell death in A549 human lung carcinoma cells [205]. Similarly, pharmacological inhibition of malonyl-CoA decarboxylase (MCD), which increased the malonyl-CoA pool, suppresses human breast cancer cell proliferation [206]. Therefore, decreasing fatty acid oxidation rates by the modulation of the malonyl-CoA pool by ACC2 and MCD suggests a potential role for these enzymes in cancer metabolism. However, ACC2 functions in other cancer types remain to be elucidated.

The role of ACC2 in obesity is more established. Skeletal muscle ACC2 phosphorylation and activity are reduced in obese patients, as a consequence of reduced AMPK activity [207, 208]. Additionally, the mRNA levels of ACC2 in white adipose tissue are lower in Zucker fatty rats than in lean rats [209]. Interestingly, the AMPK-ACC2-CPT1 axis is modulated by several adipokines, whose levels are altered in obesity. These include leptin [210], adiponectin [211], and CTRP1 [212]. Moreover, recent evidence in liver demonstrates that metformin's actions to suppress* de novo* lipogenesis and increase fatty acid oxidation require AMPK-mediated phosphorylation of ACC1 and ACC2. Thus, the significant interest in the clinical use of metformin as the therapeutic in many cancers will further contribute to the understanding of the role that ACC1/2 plays in cancer biology [213].

## 5. Conclusions

The current interest in cancer metabolism has the potential to identify common perturbations arising from differing genetic origins that may serve as therapeutic targets. As the current obesity epidemic continues to grow, there is a need to not only define cancer metabolism but also investigate how it is influenced by the obese microenvironment. It is clear that cancer fatty acid metabolism plays a significant role in cancer biology and that opportunities exist to further define this role, especially in the context of obesity-induced changes in cancer progression.

## Figures and Tables

**Table 1:** Summary of regulators of fatty acid metabolism and their effects on cancer cell biology.

Regulator of	Cancer type	Alteration	Associated outcome	Reference(s)
FA metabolism				
LPL	Prostate	Increased activity	Increased susceptibility	[17]
	Cervical	Enhanced protein expression	Increased invasion capacity	[18]
	Rectal and skin	Increased activity	Increased tumor growth	[19]
	Lung	Increased activity	Lower overall survival	[20, 21]

CD36/FAT	Colon and ovarian	Decreased gene expression	Higher metastatic capacity	[22]
	Breast	Decreased gene expression	Higher metastatic capacity	[23]

FATP	Liver	Increased gene expression	Enhanced progression	[24]

FABP4	Breast	Decreased gene expression	N/A	[25]
	Bladder	Low gene expression	Increased tumor progression and invasion capacity	[26–29]
	Prostate	Increased protein expression	Increased migration and invasion capacities	[30]
	Ovarian	Increased protein expression	Increased migration and invasion capacities	[31]

FABP5	Breast	Increased gene expression	Higher metastatic capacity and lower recurrence-free and overall survival	[32, 33]
	Endometrial	Increased gene expression	No correlated clinical outcome	[34]
	Liver and pancreatic	Increased protein expression	N/A	[35, 36]
	Prostate	Increased gene expression	Increased tumor progression	[37]
		Decreased gene expression	Increased invasion capacity and tumor growth	[26, 38, 39]

FABP7	Breast	Increased gene expression	Lower recurrence rate, improved survival	[40, 41]
		Increased nuclear localization	Increased proliferation, pleomorphism, and tumor stage	[42]
	Primary melanoma	Increased gene expression	N/A	[43]
	Renal	Increased gene expression	No correlated clinical outcome	[44, 45]

ACSL3	Glioblastoma	Increased protein expression	Increased malignant phenotype	[46]
	Colon	Increased gene and protein expression	N/A	[47]

ACSL4	Liver	Increased gene expression	Increased proliferation	[48]

ACSL5	Colon	Increased gene expression	Increased proliferation	[49]

AGPAT2	Ovarian	Increased protein and gene expression	Reduced overall survival and higher tumor grade, mitotic index, and tumor stage	[50–52]

AGPAT11	Breast, cervical, and colon	Increased gene expression	Higher tumor grade	[53]

AGPAT9	Colorectal	Increased gene and protein expression	Increased cell growth	[54]

ATGL	Lung and skin	Increased ATGL activity	Increased tumor growth and cancer-associated cachexia	[55]

HSL	Gastrointestinal	Increased gene and protein expression	Cancer-associated cachexia	[56]
	Colorectal, pancreatic, stomach, and esophageal	Increased gene expression	N/A	[57]

MAGL	Colorectal	Increased gene and protein expression	N/A	[58]
	Ovarian, breast, melanoma, and prostate	Increased gene expression	Aggressiveness	[59]

CPT1A	Ovarian	Increased gene expression	Increased tumor growth	[31]
	Breast	Increased gene and protein expression and activity	N/A	[60]
	Glioblastoma	Increased gene expression	Higher tumor grade	[61]

CPT1C	Lung	Increased gene expression	N/A	[62]
	Glioblastoma	Increased gene expression	Higher tumor grade	[61]

ACC1	Breast	Increased protein expression	Increased tumor progression	[63, 64]
	Prostate	Increased gene expression	N/A	[65]
	Lung	Decreased activity	Increased overall survival	[66]
	Liver	Increased gene expression	N/A	[67]

## Abbreviations

CD36/FAT: Cluster of differentiation 36/fatty acid translocation
LPL: Lipoprotein lipase
VLDL: Very-low density lipoprotein
FABP: Fatty acid binding protein
PPAR: Peroxisome proliferator-activated receptor
FATPs: Fatty acid transport proteins
TAG: Triacylglycerol
LPA: Lysophosphatidic acid
GPAT: Glycerol-3-phosphate acyltransferase
AGPAT: 1-Acylglycerol-3-phosphate-O-acyltransferase
DAG: Diacylglycerol
HSL: Hormone-sensitive lipase
MAG: Monoacylglycerol
MAGL: Monoacylglycerol lipase
ATGL: Adipose triglyceride lipase
ACS: Acyl-CoA synthetase
CPT1: Carnitine palmitoyltransferase 1
CoA: Coenzyme A
ETC: Electron transport chain
ACSL: Acyl-CoA synthetase long-chain family.
